# Combining Fungal Biopesticides and Insecticide-Treated Bednets to Enhance Malaria Control

**DOI:** 10.1371/journal.pcbi.1000525

**Published:** 2009-10-02

**Authors:** Penelope A. Hancock

**Affiliations:** Centre for Population Biology, Imperial College London, Silwood Park Campus, Ascot, Berkshire, United Kingdom; Swiss Tropical Institute, Switzerland

## Abstract

In developing strategies to control malaria vectors, there is increased interest in biological methods that do not cause instant vector mortality, but have sublethal and lethal effects at different ages and stages in the mosquito life cycle. These techniques, particularly if integrated with other vector control interventions, may produce substantial reductions in malaria transmission due to the total effect of alterations to multiple life history parameters at relevant points in the life-cycle and transmission-cycle of the vector. To quantify this effect, an analytically tractable gonotrophic cycle model of mosquito-malaria interactions is developed that unites existing continuous and discrete feeding cycle approaches. As a case study, the combined use of fungal biopesticides and insecticide treated bednets (ITNs) is considered. Low values of the equilibrium EIR and human prevalence were obtained when fungal biopesticides and ITNs were combined, even for scenarios where each intervention acting alone had relatively little impact. The effect of the combined interventions on the equilibrium EIR was at least as strong as the multiplicative effect of both interventions. For scenarios representing difficult conditions for malaria control, due to high transmission intensity and widespread insecticide resistance, the effect of the combined interventions on the equilibrium EIR was greater than the multiplicative effect, as a result of synergistic interactions between the interventions. Fungal biopesticide application was found to be most effective when ITN coverage was high, producing significant reductions in equilibrium prevalence for low levels of biopesticide coverage. By incorporating biological mechanisms relevant to vectorial capacity, continuous-time vector population models can increase their applicability to integrated vector management.

## Author Summary

It has recently been proposed that mosquito vectors of malaria may be controlled by biopesticide sprays containing spores of fungi that are pathogenic to mosquitoes, causing reduced blood feeding activity and eventual death. This technique has been shown to have strong potential to reduce malaria transmission rates, and may be most effective when combined with other interventions as part of an integrated vector management strategy. I develop a model to quantify the total impact of combined interventions that can affect mosquitoes at different ages and stages in their lifecycle. As a case study, I consider the combined use of fungal biopesticides and insecticide- treated bednets (ITNs), a widespread and important vector control method. The model demonstrates that these interventions combined can have strong effects on malaria transmission even in situations where each intervention acting alone has relatively little impact. In situations difficult for malaria control due to high transmission intensity and widespread insecticide resistance, the performance of the combined interventions is improved by synergistic interactions between the interventions, whereby the ITN intervention improves the performance of the fungal biopesticide intervention. The results suggest that the combined use of ITNs and fungal biopesticides may be an efficient and effective method of malaria control.

## Introduction

Malaria is a major contributer to the global disease burden, and disproportionately affects low-income countries with climates suitable for transmission [1]–[3]. Vector control strategies have proven effective in reducing malaria transmission and prevalence [4]–[6], and are a key element of current malaria control initiatives [7]–[9]. Indoor residual spraying (IRS) and insecticide-treated bednet (ITN) interventions have been and remain the dominant methods of controlling malaria vectors [4], [9]–[12], but problems of public health and insecticide resistance associated with chemical insecticides have increased interest in alternate methods, including novel biological methods [13], [14]–[16]. Because the incubation period of the malaria parasite is relatively long in comparison to the average adult mosquito lifespan, biological methods of vector control that have sublethal and lethal effects at different points in the mosquito life cycle may substantially reduce the potential for malaria transmission [17]–[21]. Such methods may be most effective when combined with established methods in a strategic manner [8],[22],[23].

In order to impact on malaria prevalence it is necessary to reduce transmission to very low levels [24],[25]. Vector management strategies that combine multiple mosquito control interventions would therefore benefit from tactical design to alter mosquito life history in ways that are likely to maximise the impact on malaria transmission, given the resources available. This paper presents an age-structured model that explores the impact of interventions that affect multiple gonotrophic and demographic processes in the mosquito on malaria transmission and prevalence. As a case study, the combined use of fungal biopesticide and ITN interventions is considered.

Biopesticides containing spores of entomopathogenic fungi are a novel strategy for controlling malaria vectors that have shown potential to cause substantial reductions in malaria transmission in laboratory and field studies [17]–[20]. The biopesticide targets adult mosquitoes, infecting them with a fungal pathogen that does not kill instantly, and can generate a wide range of mortality patterns, some early-acting while others showing a distinct delay [17],[26],[27]. Fungal pathogen-induced mortality rates typically increase with the fungal infection age, with the average times to death due to fungal infection less than 10 days [17]–[21]. This slow-acting mortality suggests that high fungal infection rates in adult mosquitoes would be required to affect malaria prevalence, however sublethal effects of fungal infection on mosquitoes have been observed which may considerably reduce their transmission potential [21]. Fungal infection can cause a reduction in the blood feeding rate and lifetime fecundity [26]. There is also evidence that co-infection with the fungal pathogen and the malaria parasite can cause greater than additive mortality and reduced transmissibility of the malaria pathogen [15],[17].

In contrast to fungal biopesticides, ITNs are an established and widely used vector control method [6], [28]–[30] that has proven successful in reducing malaria transmission and prevalence in situations where high levels of community-wide ITN coverage are achieved [6], [29]–[31]. They therefore have a focal role in current vector control initiatives [7],[10],[12]. ITNs work by targeting the adult host-seeking mosquito population, increasing the time taken for mosquitoes to find a blood meal and increasing the mortality risk while host-seeking. Both factors interact positively to reduce the likelihood that mosquitoes live long enough to contract and transmit malaria. The effect of ITNs on mosquito mortality rates depends on levels of insecticide resistance [14], [32]–[34], the persistence of the insecticide treatment, and the excito-repellency properties of the insecticide [35],[36].

This study considers mechanisms by which ITN and fungal biopesticide interventions may affect mosquito populations at the scale of the gonotrophic cycle and at within-gonotrophic cycle time scales. The gonotrophic cycle in female adult mosquitoes is often conceptualised in terms of a host-seeking stage, during which mosquitoes actively search for a blood meal, and a non-host-seeking stage, during which blood from a recent blood meal is digested, oocytes are developed and eggs are oviposited, after which host-seeking activity begins again [37],[38]. While the ITN intervention reduces the rate of host-seeking success throughout the adult mosquito population as a whole, the fungal biopesticide may also extend the host-seeking stage in fungal pathogen-infected mosquitoes due to a deterioration in flight and blood-feeding capabilities [26]. The non-host-seeking stage in fungal pathogen-infected mosquitoes may also be protracted due to impaired metabolic functioning [26]. Within a given gonotrophic cycle, the period during which mosquitoes are exposed to a risk of fungal infection may not necessarily correspond to a particular gonotrophic cycle stage. For biopesticide application as a residual treatment in and around domestic dwellings, the fungal infection risk would conceivably be higher whilst mosquitoes are host-seeking, and also for some time after they obtain a blood meal when they often rest on nearby surfaces for a period of less than 24 hours [39],[40]. In fungal pathogen-infected mosquitoes, fungal infection age, and the corresponding risk of fungal pathogen-induced mortality, increases continually, with the mortality risk for all mosquitoes being augmented during the host-seeking stage by the presence of ITNs.

Similar to [21], the population dynamic model presented here is an age-structured Susceptible-Exposed-Infectious (SEI) model based on integral equations, considering fungal pathogen-induced age-dependent mortality in adult mosquitoes that can contract the fungal infection at any point in their adult life. This model reformulates that of [21] to explicitly incorporate gonotrophic cycles in the adult mosquito population by defining a recursive series of host-seeking and non-host-seeking classes of mosquitoes. The model thus retains the properties of existing continuous [21], [41]–[43] and discrete feeding cycle approaches [31],[44],[45]. Equilibrium analysis is used to validate the model for limiting cases similar to those represented by existing continuous-time models [21],[43]. Cases where the risk of fungal infection varies throughout the gonotrophic cycle and where fungal infection causes within-population variation in the lengths of both host-seeking and non-host-seeking stages are explored numerically.

The model is parameterized with literature data on mosquito-malaria interactions, and the effects fungal biopesticides and ITNs on mosquito populations. A series of questions relevant to mosquito control by fungal biopesticides, ITNs and both interventions in combination are explored. How do sublethal effects of fungal infection on rates of finding and processing blood meals affect the impact of biopesticides on malaria transmission rates? How is the fungal biopesticide performance affected by variation in the period of biopesticide exposure within a given gonotrophic cycle? How does the performance of fungal biopesticide and ITN interventions combined compare with that of each single intervention for varying levels of transmission intensity and insecticide resistance? Mechanisms important to the performance of fungal biopesticides, ITNs and both interventions combined are identified and discussed.

## Models

**Figure 1:**
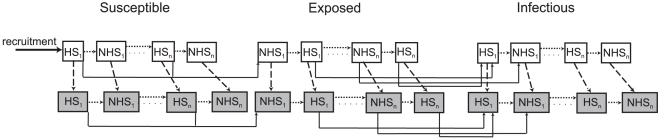
Diagram showing the progression of the mosquito population through the stages of the model. Solid lines show the malaria infection process, dashed lines show the fungal infection process and dotted lines show the gonotrophic feeding process. HS*_i_* and NHS*_i_* refer to the *i* ^th^ host-seeking and non-host-seeking stages respectively.

The dynamics of adult mosquito infection with the *Plasmodium* parasite are treated as an SEI process in continuous time with the mosquitoes being categorized as susceptible, exposed (but not yet infectious) and infectious [43]. Similar to [21], each of these three categories is divided into mosquitoes that are uninfected with the fungal pathogen and those that are infected. Mosquitoes that are infected with the fungal pathogen experience a fungal pathogen-induced mortality risk that is dependent on fungal infection age. Mosquitoes are further categorized according to their stage in the gonotrophic feeding cycle (Figure 1). Gonotrophic feeding cycle parameters may be altered by both fungal biopesticide and ITN interventions. These processes are now described in more detail and an age-structured population dynamic model is developed and analysed analytically for limiting cases. Model parameters are listed in Table 1 and model variables are listed in Table S3.

**Table 1:** Parameters used in the model.

Symbol	Definition	Value	Source
*ε*	recruitment to the adult mosquito population	0.2 (d^−1^)	
*b*	*Plasmodium* transmission probability (mosquito to human)	0.5	[43]
*b*	*Plasmodium* transmission probability (human to mosquito)	0.5	[43]
*x*	proportion of humans with transmissible malaria	0.5	[24]
*T_E_*	length of *Plasmodium* incubation period	10.0 (d)	[43]
*T_M_*	length of the non-host-seeking stage for mosquitoes not infected with the fungal pathogen	2.0 (d)	[54],[55]
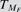	length of the non-host-seeking stage for mosquitoes infected with the fungal pathogen	2.0–4.0 (d)	
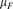	mosquito mortality (due to all factors other than fungal biopesticide and ITN interventions) during the host-seeking stage	0.1 (d^−1^)	[31],[43],[45]
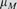	mosquito mortality (due to all factors other than fungal biopesticide and ITN interventions) during the non-host-seeking stage	0.1 (d^−1^)	[31],[43],[45]
*F*	rate of contracting fungal infection	0–11.5 (d^−1^)	
*C*	daily probability of fungal infection (1−exp(−*F*))	0–1	
*f*	rate of finding blood meals for host-seeking mosquitoes not infected with the fungal pathogen	1.2 (d^−1^)	[31]
*f_F_*	rate of finding blood meals for host-seeking mosquitoes infected with the fungal pathogen	0.11–1.2 (d^−1^)	
*P*	daily probability of finding a blood meal for host-seeking mosquitoes, given that they do not die or become infected with the fungal pathogen (1−exp(−*f*))	0.7	
*P_F_*	daily probability of finding a blood meal for host-seeking mosquitoes infected with the fungal pathogen given that they do not die (1−exp(−*f_F_*))	0.1–0.7	
*α*	proportion of the non-host-seeking stage during which mosquitoes are exposed to a risk of fungal infection	0–1	
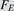	rate of contracting fungal infection throughout the portion of the gonotrophic cycle in which mosquitoes are exposed to fungal infection risk (see text)	varies (d^−1^)	
*C_E_*	daily probability of fungal infection throughout the portion of the gonotrophic cycle in which mosquitoes are exposed to fungal infection risk (see text)	varies	
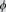	fraction of humans protected by ITNs (ITN coverage)	0–1	
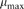	mortality rate of host-seeking mosquitoes when ITN coverage 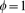	0.35 (d^−1^)	[48]

### Gonotrophic feeding processes

The blood-feeding patterns of adult female mosquitoes are assumed to follow a gonotrophic cycle whereby mosquitoes repeatedly seek a blood meal, obtain a single blood meal, and then stop seeking blood for a fixed time period after which they resume host-seeking activity and the gonotrophic cycle begins again. The model accordingly divides each gonotrophic cycle into two stages: a host seeking stage of variable duration, during which the mosquito is searching for a blood meal, and a non-host-seeking stage of fixed duration, during which the mosquito does not seek blood meals (Figure 1).

During the host-seeking stage, mosquitoes are assumed to feed soley on humans at a rate *f* (Table 1). This rate may be intuitively interpreted by the corresponding daily probability of finding a blood meal given that the mosquito does not die, 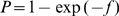 (Table 1). During the host-seeking stage the mosquito mortality rate due to all mortality sources other than fungal biopesticide and ITN interventions is 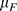. Upon finding a blood meal mosquitoes enter the non-host-seeking stage, which lasts for 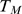 days. During the non-host-seeking stage the mosquito mortality rate due to all mortality sources other than fungal biopesticide and ITN interventions is 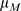. For each stage of the malaria infection process (susceptible, exposed and infectious), the maximum number of gonotrophic cycles completed, including host-seeking and non-host-seeking stages, is *n_S_*, *n_E_* and *n_I_* respectively (Table S3).

### Fungal infection

In describing the model below, the rate of fungal infection in adult mosquitoes, *F*, is assumed to be a constant. This rate may be intuitively interpreted by the corresponding daily probability of fungal infection given that the mosquito does not die, 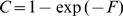 (Table 1). However fungal infection risk may differ at different points in the gonotrophic cycle and so a version of the model where *F* varies throughout the gonotrophic cycle is analysed numerically in the Results.

The mortality rate of mosquitoes that are infected with the fungal pathogen is increased by an amount 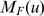 that may vary with *u*, the time that has elapsed since infection [21]. As in [21], 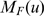, is modelled using the Weibull model 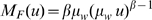, where 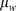 and *β* are the rate and shape parameters respectively (Table S3). The measure of fungal pathogen virulence is the expected time until death due to fungal infection given no other mortality, 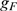, given by
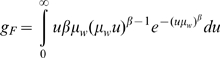
(1)
[21].

Sublethal effects of fungal infection on mosquitoes are also considered, including a reduction in the host-seeking success and an increase in the time to the next host-seeking period following a blood meal in fungal pathogen-infected mosquitoes. This is modeled by a reduced blood feeding rate 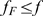 and an increased duration of the non-host-seeking stage 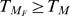 in fungal pathogen-infected mosquitoes (Table 1).

### Insecticide treated bednets (ITNs)

Similar to [45], an ITN intervention is assumed to affect two gonotrophic cycle parameters, the mortality rate of host-seeking mosquitoes, 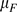, and the rate at which host-seeking mosquitoes take blood meals, *f_F_* or *f* for mosquitoes with and without fungal infection respectively. The blood-feeding rates *f_F_* and *f* are multiplied by a factor 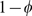, where 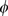 is the fraction of humans protected by ITNs [45]. This assumes that mosquitoes do not bite ITN users or non-human hosts. The parameter 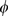 is referred to as the ITN coverage, in line with ITN literature [6],[31],[45]. The mortality rate 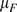 is assumed to be directly proportional to 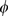 such that 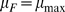 when 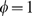 (Table 1). The effect of ITNs on mosquito mortality can be reduced by insecticide resistance in the mosquito population [32],[34], limited persistence of the insecticide treatment, or excito-repellency properties of the insecticide [36]. Therefore, the limiting case in which the ITN intervention has no effect on mosquito mortality is also considered.

### Model formulation

The model is described by a system of integral equations for the mosquito density, defined as the number of mosquitoes per human [21],[43], as a function of time in each of a series of 12 stages. The stages correspond to the mosquito's malaria infection status (susceptible, exposed, infectious), gonotrophic cycle stage (host-seeking, non-host-seeking), and fungal infection status (infected, uninfected). Equations for stages of mosquitoes without fungal infection (Text S1) are considered separately from stages of fungal pathogen-infected mosquitoes (Text S2). A series of expressions are defined, 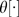, which give the probability that a mosquito remains in the same stage over different periods of time. These are described in more detail in Text S1 and Text S2 and are listed in Tables S1 and S2. Similar to [21],[43], the model assumes that mosquitoes are recruited to the adult population at a constant rate *ε*, that a constant fraction, *x*, of the human population is infected with malaria, the probability of transmission from an infected human or an infected mosquito is *b*, and that it takes the *Plasmodium* exactly *T_E_* days to mature in mosquito and become infectious (Table 1).

### Equilibrium analysis

The equilibrium daily EIR, denoted 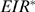, is given by
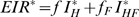
(2)where 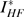 and 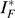 are the equilibrium densities of infectious, host-seeking mosquitoes with and without fungal infection respectively. To qualitatively estimate the relationship between the model-derived equilibrium daily EIR and the malaria prevalence in the human population, denoted 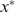, the best fit model obtained by [25] was used, with no change to the best fit parameters. This provides a conservative estimate of the prevalence, because the equilibrium EIR estimates from this model do not take into account reductions in prevalence that may occur as a result of the decrease in EIR resulting from fungal biopesticide and ITN interventions.

**Figure 2:**
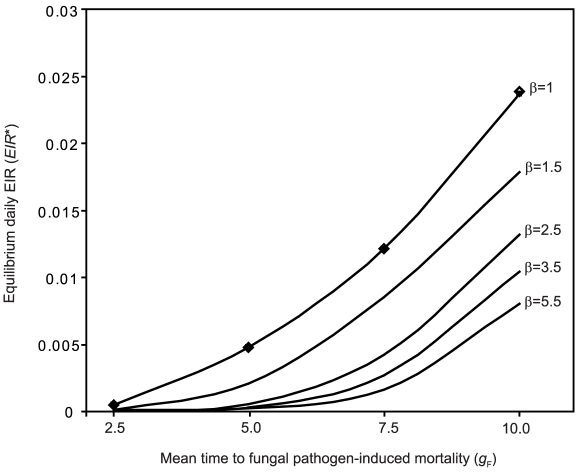
Comparing equilibria obtained by simulation and analytic derivation. Equilibrium daily EIR as a function of the mean time to death if the fungus is the only mortality source (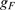) for different values of the shape parameter 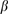 and other parameters as in Table 1. For 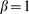 values of the equilibrium daily EIR obtained by analytic derivation (open diamonds) and simulation (black squares) are compared. Sublethal effects of fungal infection on mosquito feeding biology are not incorporated (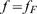 & 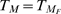).

Expressions are derived for the equilibrium density of susceptible, exposed and infectious host-seeking mosquitoes for the limiting case in which there are no sublethal effects of fungal infection on mosquito feeding biology (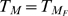, 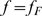) and the shape parameter of the fungal pathogen-induced mortality function 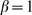 (Text S1 and Text S2). The analytically derived equilibrium EIR agrees well with the equilibrium obtained by simulating equations (S1.1)–(S1.17) and (S2.1)–(S2.18) through time using a simulation algorithm coded in C++ (Figure 2). The simulation algorithm is used to obtain the equilibrium for the general case in which 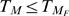, 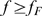 and 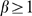. The pattern in Figure 2 is similar to that produced by simpler models [21], whereby increasing the shape parameter *β* above 1 reduces the equilibrium malaria transmission rate.

**Figure 3:**
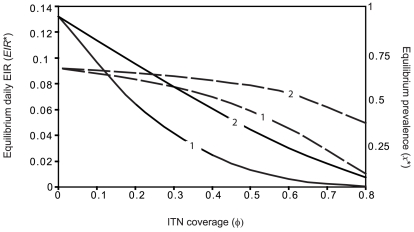
Equilibrium daily EIR and prevalence when the ITN intervention is applied in the absence of the biopesticide. Equilibrium daily EIR (solid lines) and equilibrium human prevalence (dashed lines) as a function of ITN coverage (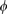). Lines labelled 1 correspond to the ITN intervention parameters in Table 1, and lines labelled 2 correspond to the case where the ITN intervention has no effect on mosquito mortality (other ITN intervention parameters are as in Table 1).

The ITN intervention corresponds to a limiting case of the model in which the fungal infection rate *F* is zero. For this case, the dynamic system is much simpler, requiring only equations (S1.1)–(S1.17), and solution of the equilibrium EIR is much simpler, given by equations (S1.18)–(S1.27). The ITN intervention produces a rapid decline in the equilibrium EIR as the ITN coverage (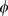) increases, but does not have a strong impact on the equilibrium malaria prevalence in humans until the level of ITN coverage is moderate to high (Figure 3). These patterns are similar to those produced by [31],[45]. Figure 3 also shows the case where the ITN intervention has no effect on mosquito mortality, and thus affects only the blood feeding rates *f_F_* and *f*. This may represent widespread insecticide resistance in the mosquito population.

### Model parameterisation

The aim of the models developed here is to explore generic issues relating to fungal biopesticide and ITN interventions rather than to parameterise a specific case. In order to use biological relevant parameters, a survey of the literature was conducted (Table 1). Using these parameters, the model gives an equilibrium annual EIR in the absence of the fungal biopesticide and ITNs of 47.8, which consistent with a number of regions in Africa with moderate to high malaria prevalence [24],[25]. This is similar to the value of 45 given by the continuous time models of [21],[43] with a human biting rate of 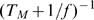 and other parameters as in Table 1. Similar to [21], the equilibrium baseline EIR scales linearly with the recruitment rate *ε*.

## Results

The model was explored by asking a series of questions relevant to mosquito control by fungal biopestides, ITNs and the combined use of both these interventions, using the equilibrium EIR and the corresponding estimate of human malaria prevalence as a measure of successful intervention.

### How do sublethal effects of fungal infection on mosquito blood-feeding biology influence the effect of fungal biopesticides on malaria transmission rates?

**Figure 4:**
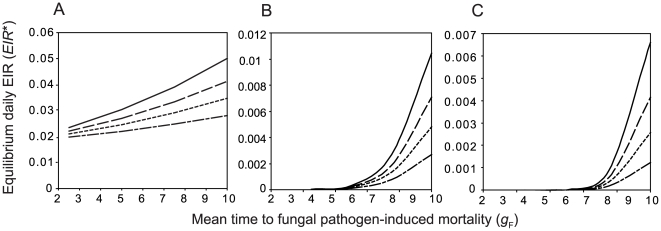
The impact of sublethal effects of fungal infection on mosquito feeding biology. Equilibrium daily EIR as a function of the mean time to death from fungal infection (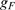) for three values of the daily probability of fungal infection (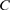): A, *C* = 0.1 B, *C* = 0.5 C, *C* = 0.9. Lines represent varying sublethal effects of fungal infection on mosquito feeding biology, including no sublethal effects (solid lines), a 25% decrease in the daily probability of finding a blood meal (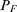) and a 25% increase in the duration of the non-host-seeking stage (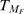) in fungal pathogen-infected mosquitoes (dashed lines), a 50% decrease in 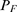 and a 50% increase in 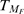 (dotted lines), and a 75% decrease in 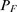 and a 75% increase in 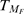 (dot-dashed lines). Other parameters are given in Table 1.

Fungal infection can substantially reduce mosquito blood-feeding activity [26]. Here, two possible effects of fungal infection on mosquito blood-feeding biology are considered, including a reduction in the blood-feeding rate in host-seeking mosquitoes, 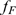, and an increase in the duration of the non-host-seeking stage, 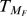 (Table 1). Even when these effects act simultaneously, they have less potential to produce very low equilibrium EIR than decreasing the average time to death from fungal infection, 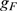 (Figure 4). The strongest sublethal effects shown in Figure 4 represent more than a four fold increase in the average gonotrophic cycle length in fungal pathogen-infected mosquitoes, 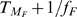. For moderate to high daily probability of fungal infection, this has a similar effect to a 25% reduction in the average time to death from fungal infection (Figure 4B and C). When the daily probability of fungal infection is low, reductions in the equilibrium EIR obtained by either increasing the fungal pathogen virulence (by reducing 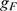) or increasing the sublethal effects are considerably less, and the impact of strong sublethal effects on the EIR is of similar magnitude to that produced by strong reductions in 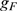 (Figure 4A).

### How is fungal biopesticide performance affected by varying fungal infection risk throughout the gonotrophic cycle?

For a fungal biopesticide applied in and around human settlements, mosquitoes may not be exposed to a risk of fungal infection for their entire gonotrophic cycle. They may be most likely to contract the fungal pathogen when they are host seeking and for a time period directly after blood feeding. A shorter period of exposure to a risk of fungal infection within a given gonotrophic cycle may lead to lower fungal infection levels in the mosquito population, with implications for fungal biopesticide performance. Here, the effect of varying fungal infection risk throughout the gonotrophic cycle is explored by varying the fungal infection rate, *F*, such that non-host-seeking mosquitoes experience a constant fungal infection rate 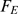 for an initial proportion of the non-host-seeking stage, denoted *α*, and a fungal infection rate of zero for the remainder of the non-host-seeking stage (Table 1). The corresponding daily probability of fungal infection during the period of biopesticide exposure is 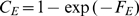 (Table 1). Host-seeking mosquitoes are assumed to experience fungal infection rate 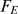 (and daily probability of fungal infection *C_E_*) throughout the entire host-seeking stage.

**Figure 5:**
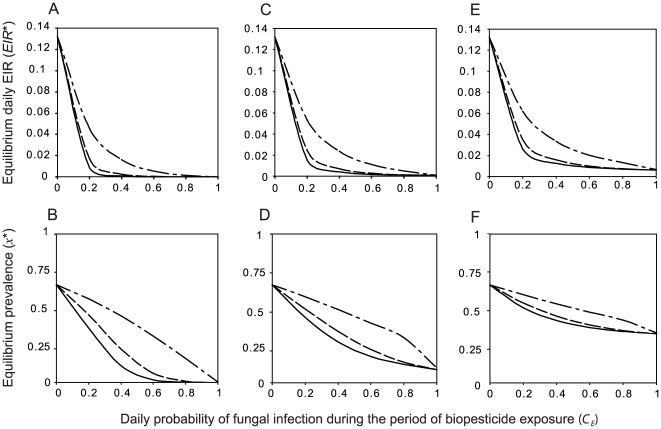
The effect of varying the period of biopesticide exposure in a given gonotrophic cycle. Equilibrium daily EIR and equilibrium human prevalence as a function of the daily probability of fungal infection during the period of biopesticide exposure (*C_E_*) for three values of the mean time to death from fungal infection (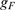): A–B. 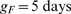, C–D. 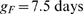, E–F. 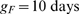. Lines represent biopesticide exposure periods (see text) corresponding to 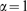 (solid lines), 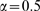 (dashed lines) and 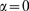 (dot-dashed lines).

The equilibrium daily EIR is marginally higher if mosquitoes are exposed to fungal infection risk for half of the non-host-seeking stage (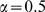) in comparison to exposure for the full stage duration (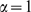) (Figure 5A, C, E). However, if mosquitoes are only exposed to fungal infection risk when they are host-seeking (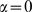), the equilibrium EIR is considerably higher. The corresponding estimate of the equilibrium malaria prevalence in humans varies more between the three exposure periods than the equilibrium EIR (Figure 5B, D, F), because it is sensitive to changes in EIR at low EIR values. The effect of varying the period of biopesticide exposure on prevalence is greater for more virulent biopesticides, which benefit more from the very low transmission levels achieved for longer duration of exposure to fungal infection risk (Figure 5A, B). Similar to [21], if mosquitoes are always exposed to fungal infection risk, there is a threshold level of the daily probability of fungal infection above which additional reductions in equilibrium EIR and prevalence are marginal. This is not the case if mosquitoes are only exposed to fungal infection risk when they are host-seeking, where prevalence continues to decline steadily as the daily probability of fungal infection is increased to high values (Figure 5).

### How does the combined use of fungal biopesticides and insecticide treated bednets (ITNs) affect malaria control in comparison to each single intervention for varying levels of intervention coverage?

ITNs can be an effective means of malaria control if high levels of community-wide ITN coverage can be achieved [31]. Fungal biopesticides may be used in combination with ITN interventions at varying levels of coverage to give greater reductions in malaria transmission and human prevalence. To quantify this effect, the impact of biopesticide application on the equilibrium EIR and human malaria prevalence is explored for fixed levels of ITN coverage 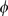. The daily probability of fungal infection during the period of biopesticide exposure (*C_E_*) is referred to as the fungal biopesticide coverage throughout this section. The conservative assumptions that mosquitoes are only exposed to a risk of fungal infection during the host-seeking stage (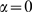), at a constant rate 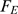, and that there are no sublethal effects of fungal infection on mosquito feeding biology (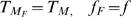) are adopted (Table 1).

**Figure 6:**
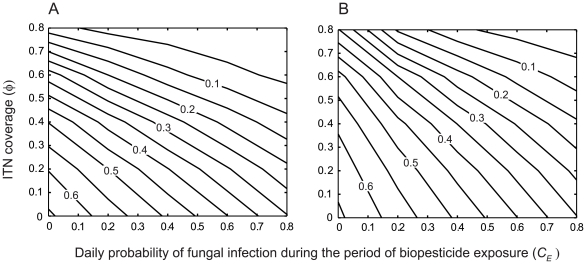
The combined impact of fungal biopesticides and ITNs on human malaria prevalence. Isolines of the equilibrium human prevalence (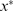) for varying ITN coverage (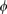) and daily probability of fungal infection during the period of biopesticide exposure (*C_E_*), for different ITN intervention parameters: A. The ITN intervention parameters given in Table 1, B. The ITN intervention does not affect mosquito mortality (other ITN intervention parameters are as in Table 1). The mean time to death from fungal infection is 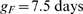.

**Figure 7:**
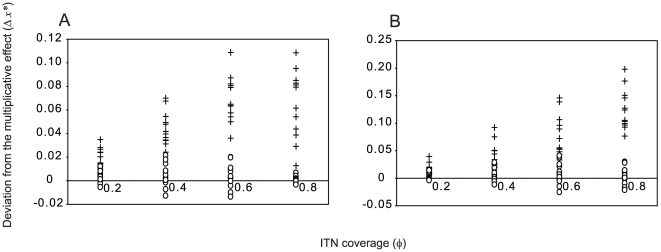
Comparing the impact of the combined interventions to the multiplicative effect of both interventions. The amount by which the equilibrium prevalence deviates from the multiplicative effect of both interventions (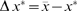) as a function of the ITN coverage (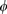). Points represent different ITN intervention parameters, including the parameters given in Table 1 (open circles) and the case where the ITN intervention does not affect mosquito mortality (crosses). All combinations of mean times to death from fungal infection (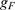) of 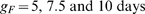, and values of the daily probability of fungal infection during the period of biopesticide exposure (*C_E_*) of *C_E_* = 0.2, 0.4, 0.6 and 0.8, are shown. Panels show different levels of transmission intensity: A. Annual EIR = 47.8, B. Annual EIR = 412.

Low values of equilibrium prevalence are not obtained by the fungal biopesticide intervention alone, or by the ITN intervention alone unless ITN coverage is high (Figure 6A). When both interventions are used in combination, low prevalence is obtained with moderate coverage of each intervention. The proportional reduction in equilibrium EIR obtained by the combined interventions found to be very close to the multiplicative effect of both interventions, or the product of the proportional reductions given by each intervention acting alone. The difference between the equilibrium prevalence obtained from the combining the two interventions, 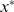, and the prevalence corresponding to the multiplicative effect of the two interventions on the equilibrium EIR, denoted 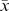, is calculated as 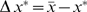. As 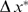 is small in this case (Figure 7A, open circles), there is negligible redundancy in combining both interventions, and also negligible synergistic effects, or no increase in the impact of one intervention on malaria transmission due to the presence of the other intervention.

### How does insecticide resistance affect the performance of fungal biopesticide and ITN interventions used in combination?

The mortality in host-seeking mosquitoes caused by the ITN intervention may be reduced by the development of insecticide resistance in the mosquito population. To give a conservative estimate of intervention performance, the limiting case in which the ITN intervention causes no increase to mosquito mortality is examined. In this case the ITN intervention affects only the blood-feeding rate of host-seeking mosquitoes, by providing a physical barrier between mosquitoes and the human hosts that are protected. For this scenario, low values of equilibrium prevalence are still be obtained by the ITN and fungal biopesticide interventions combined, although higher coverage of each intervention is required (Figure 6B). The baseline equilibrium prevalence is reduced by approximately 50% by the two interventions combined with moderate coverage of each intervention, whereas a similar reduction requires high coverage of each intervention acting alone (Figure 6B).

When the ITN intervention has no mortality effect on mosquitoes, the reduction in equilibrium prevalence obtained by the interventions combined is greater than the multiplicative effect of both interventions (Figure 7A, crosses). The deviation from the multiplicative effect generally increases with increasing ITN coverage (Figure 7A), although not with increasing fungal biopesticide coverage (results not shown). Thus when ITN coverage is high, the addition of the fungal biopesticide has a large impact on prevalence even at low biopesticide coverage (Figure 6B). The combined effect of the two interventions, being greater than multiplicative, is indicative of synergistic interactions between the interventions. This synergism results from the increase in the average time required for mosquitoes to find a blood meal due to the presence of non-lethal bednets, which increases the period of exposure to the fungal biopesticide in a given gonotrophic cycle, and improves the performance of the fungal biopesticide intervention.

### How does variation in malaria transmission intensity affect the performance of fungal biopesticide and ITN interventions used in combination?

Variation in malaria transmission intensity will affect the efficacy of vector control strategies and may alter the appropriate choice of strategy. The case of high malaria transmission intensity is considered here by increasing the recruitment rate *ε* to give an annual EIR of 412. This value is similar to levels of transmission measured in the high transmission season in the Garki district of Nigeria, an area where malaria control has proven difficult [46].

**Figure 8:**
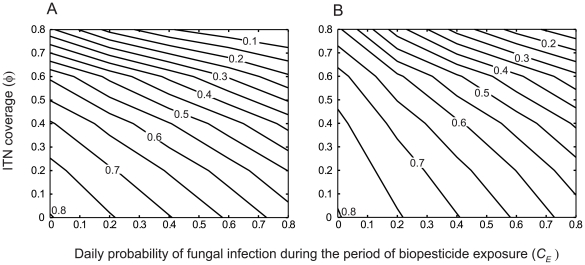
The effect of the combined interventions on human prevalence for high transmission. Isolines of the equilibrium human prevalence (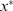) for varying ITN coverage (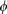) and daily probability of fungal infection during the period of biopesticide exposure (*C_E_*), for different ITN intervention parameters: A. The ITN intervention parameters given in Table 1, B. The ITN intervention does not affect mosquito mortality (other ITN intervention parameters are as in Table 1). The transmission intensity is high (annual EIR = 412). The mean time to death from fungal infection is 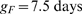.

When transmission intensity is high, low values of equilibrium prevalence are not obtained by either the fungal biopesticide or the ITN intervention acting alone, but are obtained when ITN and fungal biopesticide interventions are combined if ITN coverage is high and fungal biopesticide coverage is moderate (Figure 8A). When ITN coverage is high, fungal biopesticide application is again more effective, producing a sharp decline in prevalence even at low biopesticide coverage. For high transmission intensity, the combined effect of the two interventions is similar to the multiplicative effect of both interventions (Figure 7B, open circles)

As above, the ITN intervention is now considered to have no effect on mosquito mortality, representing widespread insecticide resistance. In this case, considerable reductions in prevalence are only obtained when ITN and fungal biopesticide interventions are combined (Figure 8B). Low equilibrium prevalence is still obtained by the combined interventions for high levels of coverage of each intervention. When the ITN intervention does not affect mosquito mortality, the combined effect of the two interventions exceeds the multiplicative effect of both interventions, to a greater extent than when transmission intensity was lower (Figure 7B, crosses). This demonstrates a case of considerable synergism between the two interventions, whereby the ITN intervention improves the performance of the fungal biopesticide intervention, particularly when ITN coverage is high (Figure 8B).

**Figure 9:**
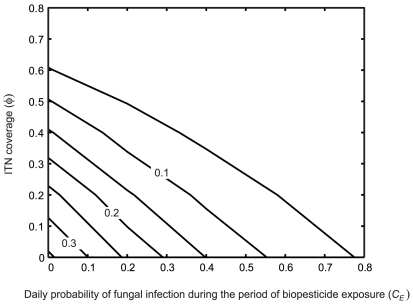
The effect of the combined interventions on human prevalence for low transmission. Isolines of the equilibrium human prevalence (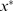) for varying ITN coverage (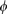) and daily probability of fungal infection during the period of biopesticide exposure (*C_E_*). The transmission intensity is low (annual EIR = 1.5). The mean time to death from fungal infection is 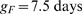.

Low transmission intensity is simulated by decreasing *ε* to give an annual EIR of 1.5, a value in the lower part of the range reported in [25]. In this case, low values of equilibrium prevalence are obtained by the combined use of fungal biopesticide and ITN interventions for low to moderate levels of coverage of each intervention. If the interventions act alone the level of coverage required to achieve low prevalence is considerably higher, though still moderate (Figure 9).

## Discussion

This study models the effect of fungal biopesticide interventions on rates of malaria transmission with greater biological detail than [21]. While the models are not formally equivalent, the results of this model are consistent with those of [21] for the limiting case in which mosquitoes are continually exposed to a constant risk of fungal infection and there are no sublethal effects of fungal infection on the mosquito blood-feeding biology. The baseline EIR (in the absence of the interventions) is also similar for the two models. The same assumptions about mosquito-fungus interactions described in [21] apply to this model with two exceptions. Firstly, by incorporating gonotrophic structure, this model can consider the effects of fungal infection on host-seeking and non-host-seeking stages of the gonotrophic cycle. Secondly, this model allows the fungal infection rate to vary throughout the gonotrophic cycle.

Firstly, the model results indicate that high virulence will be important to the success of the fungal biopesticide intervention even if fungal infection has a strong effect on both the average time taken for host-seeking mosquitoes to find a blood meal and the time to the next period of host-seeking activity following a blood meal. Secondly, assuming realistically that mosquitoes are not exposed to the fungal biopesticide for their entire gonotrophic cycle, the results indicate that a high daily probability of fungal infection during the period of biopesticide exposure will be important to the success of the biopesticide intervention. This implies an important role for strategies designed to prolong the period of biopesticide exposure, such as the use of African water storage pots sprayed with the biopesticide [20].

Consistent with the goals of Integrated Vector Management frameworks [7],[8],[10], the model explores the total impact of multiple mosquito control interventions used in combination. The principle and practise of combining interventions to give substantial impacts on malaria transmission and manage insecticide resistance is not new [5],[8],[41],[47],[48]. This study explores interactions not previously considered, namely those between ITNs, an widely-used method of controlling malaria vectors, and fungal biopesticides, a novel method of biocontrol that has slow-acting and potentially complex effects on the mosquito life cycle.

In the absence of field data on the combined application of fungal biopesticides and ITNs, conservative assumptions were adopted. Fungal infection was assumed to affect only mosquito mortality, and the exposure of mosquitoes to both interventions was restricted to the host-seeking stage of the gonotrophic cycle. Similar to [31],[49], the equilibrium EIR estimates given by this model are conservative in that they do not take into account changes in human malaria prevalence that may result from the interventions. This assumption may be accurate in the short term given that the lifespan of the vector is considerably shorter than the duration of malaria infection in humans [25]. This SEI model could be extended incorporate human prevalence dynamics [50], however the relationship between human prevalence and rates of malaria transmission is known to be complex and heterogeneous [25].

Low estimates of the equilibrium prevalence were obtained by combining fungal biopesticide and ITN interventions even for scenarios where the impact of each intervention acting alone was relatively small, indicating that the combination is effective. In general, the impact of combining the two interventions on malaria transmission was at least as good as the multiplicative effect of both interventions, which intuitively demonstrates that the combination is efficient. This need not be the case, for example, if large numbers of fungal pathogen-infected mosquitoes are killed by ITNs, the effective coverage of the fungal biopesticide would be reduced. Figure 7 shows that the impact of the combined interventions is sometimes slightly less than multiplicative, which is indicative of this effect. A spatially heterogeneous process, whereby locations that are sprayed with the fungal biopesticide are also those that are best protected by ITNs, may lead to correlation between the probability of fungal infection and the probability of encountering an ITN. This may result in greater redundancy in the combined effect of both interventions compared to the multiplicative effect.

However, as conditions for malaria control become more difficult due to increasing transmission intensity or the development of insecticide resistance, interactions between the two interventions become increasingly synergistic, in that the performance of the fungal biopesticide is enhanced by the ITN intervention. This allowed low prevalence to be obtained for the combined interventions even for high transmission intensity and widespread resistance of mosquitoes to mortality from ITNs. The mechanism underlying this synergism, namely the protraction of the period of exposure to the fungal biopesticide due to the presence of non-lethal ITNs, may be more robust to spatial heterogeneity in the application of both interventions. Deflection of mosquitoes by non-lethal bednets may increase their likelihood of encountering the biopesticide whether it is sprayed at the same location or at another location within the range of mosquito diffusion.

The model results suggest that combining fungal biopesticide and ITN interventions can allow each intervention to be used at lower coverage to maintain a given level of malaria control. This may increase the persistence of each intervention, as lower coverage of each intervention may reduce the selection pressure for the evolution of resistance. However, cautious interpretation is again necessary. The mechanisms behind the success of high community-wide ITN coverage observed in the field may be more complex than those represented by homogenous models of ITN interventions, including the model presented here. Evidence that malaria transmission in human populations is highly heterogenous supports this suggestion [25]. It is encouraging, however, that if high ITN coverage is achieved, the model results indicate that fungal biopesticide application can be very effective even at low biopesticide coverage, particularly when transmission intensity is high and insecticide resistance is widespread.

The model presented here could also be extended to incorporate additional gonotrophic processes with important malaria transmission implications. Firstly, multiple blood feeding within a single gonotrophic cycle could considerably alter transmission patterns, depending on the mosquito life-stages in which it occurs. Multiple blood meals per cycle may be more common in newly emerged *Anopheles* mosquitoes [51], but can also be more prominent in mosquitoes harbouring infectious sporozoites [52], with the later having the most serious epidemiological implications. Secondly, the time required for blood meal digestion and oocyte development in mosquitoes decreases with increasing temperature, as does the *Plasmodium* incubation period, leading to substantial variation in these parameters across different field locations [38],[53]. Covariation in duration of the non-host-seeking stage and the *Plasmodium* incubation period may have a stronger effect on malaria transmission compared to varying each parameter in isolation.

By quantifying the impact of the combined use of fungal biopesticide and ITN interventions on malaria transmission and prevalence, the model indicates that these interventions combined may considerably improve malaria control even in situations each single intervention would have a relatively low impact. Modelling is no substitute for field studies, and attempts to make generalizations about vector biology need to be cautiously interpreted [37]. Recent vector control initiatives encourage the development of models that have the capacity to use field data to guide decision making [7]. This study demonstrates that biological mechanisms relevant to vectorial capacity can be built into existing continuous-time, population-level frameworks to allow direct parameterization from field and laboratory data on both established and novel interventions. This is a means by which models can increase their applicability to integrated vector management strategies.

## Supporting Information

Text S1 Dynamic system and equilibria for stages of mosquitoes uninfected with the fungal pathogen.
(0.35 MB DOC)

Text S2 Dynamic system and equilibria for stages of mosquitoes infected with the fungal pathogen.
(0.28 MB DOC)

Table S1 The functions,.θ[·], for the probabilities that mosquitoes remain in a given stage for a certain time period, for stages of mosquitoes uninfected with the fungal pathogen.
(0.06 MB DOC)

Table S2 The functions,.θ[·], for the probabilities that mosquitoes remain in a given stage for a certain time period, for stages of mosquitoes infected with the fungal pathogen.
(0.07 MB DOC)

Table S3 Model variables.
(0.05 MB DOC)
