# Supplementary material for: Combining Fungal Biopesticides and Insecticide-Treated Bednets to Enhance Malaria Control
Source: PLoS Comput Biol. 2009 Oct 2;5(10):e1000525. doi: 10.1371/journal.pcbi.1000525 (PMC2742557; doi:10.1371/journal.pcbi.1000525)
Supplement: Text S1 — Dynamic system and equilibria for stages of mosquitoes uninfected with the fungal pathogen. (0.35 MB DOC) [file pcbi.1000525.s001.doc]

Text S1: Dynamic system and equilibria for stages of mosquitoes uninfected with the fungal pathogen

A system of integral equations for the mosquito density as a function of time in each of a series of different stages is described below. The stages correspond to the mosquito’s malaria infection status (susceptible, exposed, infectious), gonotrophic cycle stage (host-seeking, non-host-seeking) and fungal infection status (uninfected, infected). Here, stages of mosquitoes uninfected with the fungus are considered. Stages with mosquitoes that are infected with the fungal pathogen are considered in Text S2. A series of expressions, $\theta[\cdot]$, which give the probability that a mosquito remains in the same stage over different periods of time are listed in Table S1. Variables used in the model are listed in Table S3.

*(i) Mosquito population dynamics*

*Susceptible mosquitoes*

Begin with susceptible mosquitoes that are uninfected with the fungal pathogen and are searching for a blood meal, and therefore in the host-seeking stage of the gonotrophic cycle. Mosquitoes can complete a maximum of $n_{S}$ gonotrophic cycles (including host-seeking and non-host-seeking stages) whilst they remain in the susceptible stage (Table S3). Thus let $S_{H_{i}}(t)$ denote the density of mosquitoes that are susceptible, uninfected with the fungal pathogen, and in the *i^th^* host-seeking stage, $i=1,\ldots,n_{S}$, at time *t*. The first host-seeking stage (*i*=1) consists of all mosquitoes that have recruited into the class up until the present time multiplied by $\theta_{S_{H}}(\xi_{1})$, the probability of remaining in the stage for $\xi_{1}$days (Table S3) from the time of recruitment *t-*$\xi_{1}$ to the present. The $\theta_{S_{H}}(\xi_{1})$ term encompasses the probability of not dying, not contracting the fungus, and not finding a blood-meal in time $\xi_{1}$ and is defined in Table S1.

$S_{H_{1}}(t)=\int_{0}^{t} \varepsilon\theta_{S_{H}}(\xi_{1})d\xi_{1}$ (S1.1)

Mosquito densities in host-seeking stages $i>1$ at time *t* include those that entered the *i^th^* host-seeking stage at all times *t-*$\xi_{i}$, from time (*i-*1)*T_M_* to the present, and survived for $\xi_{i}$ days. These were in host-seeking stage *i*-1 at time *t-*$\xi_{i}$*- T_M_* and found a blood meal at that time and did not contract the *Plasmodium.* They then survived the *i*-1^th^ non-host-seeking period, lasting *T_M_* days, without contracting the fungus, with probability $\theta_{S_{M}}(T_{M})$ (Table S1). The probability of then surviving in the *i^th^* host-seeking stage for $\xi_{i}$ days without finding a blood meal or contracting the fungus is $\theta_{S_{H}}(\xi_{i})$ (Table S1). Thus,

$S_{H_{i}}(t)=\int_{0}^{t-(i-1)T_{M}} S_{H_{i-1}}(t-\xi_{i}-T_{M})f(1-bx)\theta_{S_{M}}(T_{M})\theta_{S_{H}}(\xi_{i})d\xi_{i} i>1$ (S1.2)

Now consider non-host-seeking mosquitoes, which can complete a maximum of $n_{S}$ non-host-seeking stages whilst in the susceptible stage. The density of mosquitoes that are uninfected with the fungal pathogen and in their *i^th^* non-host-seeking stage is denoted $S_{M_{i}}(t)$. These mosquitoes found a blood meal in the *i*^th^ host-seeking stage at time *t-a* (Table S3) and did not contract the *Plasmodium.* They then survived in the *i*^th^ non-host-seeking stage without contracting the fungus or finding a blood meal for time *a* <*T_M_* with probability $\theta_{S_{M}}(a)$ (Table S1)*.*  Therefore,

$S_{M_{i}}(t)=\int_{0}^{T_{M}} S_{H_{i}}(t-a)f(1-bx)\theta_{S_{M}}(a)da$ (S1.3)

The total densities of susceptible mosquitoes uninfected with the fungal pathogen in each stage of the gonotrophic cycle at time *t* are denoted $S_{F}(t)$and $S_{M}(t)$ for host-seeking and non-host-seeking stages respectively.

$S_{H}(t)=\sum_{i}^{n_{S}} S_{H_{i}}(t)$ (S1.4)

$S_{M}(t)=\sum_{i}^{n_{S}} S_{M_{i}}(t)$ (S1.5)

*Exposed mosquitoes*

Susceptible mosquitoes that take a blood meal from a malaria-infectious human and contract the malaria parasite enter the exposed stage. These mosquitoes can complete a maximum of $n_{E}$gonotrophic cycles (including host seeking and non-host-seeking stages) whilst they remain in the exposed stage (Table S3). For exposed mosquitoes, we need to keep track of the length of time they have carried the *Plasmodium, p* (Table S3). Thus let $E_{H_{i}}(t,p)$denote the density of exposed mosquitoes that are uninfected with the fungal pathogen and in host-seeking stage *i, $i=1,...,n_{E}$,* with *Plasmodium* infection age *p* (Table S3)*.* The first host-seeking stage, $E_{H_{1}}(t,p)$, consists of those susceptible mosquitoes uninfected with the fungus that found a blood meal and contracted the *Plasmodium* at time *t-p,* then survived the following non-host-seeking period without contracting the fungal pathogen, with probability $\theta_{E_{M}}(T_{M})$(Table S1). They then survived for *p-*$T_{M}$ days in the first host-seeking stage of the exposed stage without finding a blood meal or contracting the fungal pathogen, with probability $\theta_{E_{H}}(p-T_{M})$(Table S1). Thus

$E_{H_{1}}(t,p)=S_{H}(t-p)fbx\theta_{E_{M}}(T_{M})\theta_{E_{H}}(p), T_{M}\leq p\leq T_{E}$ (S1.6)

For host-seeking stages $i>1$, $E_{H_{i}}(t,p)$ consists of mosquitoes from *i*-1^th^ host-seeking stage, uninfected with the fungal pathogen, that found a blood meal at time $t-T_{M}-\xi_{i}$ when their *Plasmodium* infection age was $p-T_{M}-\xi_{i}$*,* where $\xi_{i}$is the time spent in the *i*^th^ host-seeking stage (Table S3). These mosquitoes avoided both mortality and fungal infection in the *i*^th^ non-host-seeking period with probability $\theta_{E_{M}}(T_{M})$(Table S1), and then in the *i*^th^ host-seeking period without finding a blood meal, with probability $\theta_{E_{H}}(\xi_{i})$ (Table S1).

$$E_{H_{i}}(t,p)=\int_{0}^{p-iT_{M}} E_{H{}_{i-1}}(t-\xi_{i}-T_{M,}p-T_{M}-\xi_{i})f\theta_{E_{M}}(T_{M})\theta_{E_{H}}(\xi_{i})d\xi_{i} i>1,iT_{M}\leq p\leq T_{E}$$

(S1.7)

Now consider exposed, non-host-seeking mosquitoes, in each non-host-seeking stage *i*, $i=1,...,n_{E}$. For mosquitoes uninfected with the fungal pathogen, specify the *Plasmodium* infection age *p* and the time since the most recent blood meal, *a* , $0\leq a\leq T_{M}$(Table S3). For the first non-host-seeking stage (*i*=1), the mosquito density, denoted $E_{M_{1}}(t,p,a)$, consists of susceptible, host-seeking mosquitoes uninfected with the fungal pathogen that found a blood meal at time *t-a*, contracted malaria, and then survived *a* days in the first non-host-seeking stage without contracting the fungus.

$E_{M_{1}}(t,p,a)=S_{H}(t-a)fbx\theta_{E_{M}}(a) ,0\leq p<T_{M}$ (S1.8)

For the mosquito density in non-host-seeking stages *i*>1, denoted $E_{M_{i}}(t,p,a)$, mosquitoes come from host-seeking stage *i*-1, having found a blood meal at time *t-a* when their *Plasmodium* infection age was *p-a,* and then survived *a* days in the *i*^th^ non-host-seeking stage without contracting the fungus.

$E_{M_{i}}(t,p,a)=E_{H_{i-1}}(t-a,p-a)f\theta_{E_{M}}(a) ,(i-1)T_{M}+a\leq p\leq T_{E}, i>1$ (S1.9)

Total densities of exposed mosquitoes uninfected with the fungal pathogen are denoted as $E_{H}(t)$and $E_{M}(t)$ for host-seeking and non-host-seeking stages respectively.

$E_{H}(t)=\sum_{i}^{n_{E}} \int_{0}^{T_{E}} E_{H_{i}}(t,p)dp$ (S1.10)

$E_{M}(t)=\sum_{i}^{n_{S}} \int_{0}^{T_{M}} \int_{0}^{T_{E}} E_{M_{i}}(t,p,a) dp da$ (S1.11)

*Infectious mosquitoes*

Mosquitoes enter the infectious stage when their *Plasmodium* infection age reaches the length of the incubation period $T_{E}$ (Table 1). Infectious mosquitoes can complete a maximum of $n_{I}$gonotrophic cycles, including host-seeking and non-host-seeking stages. First calculate the density of infectious mosquitoes uninfected with the fungal pathogen that are in the *i*^th^ host-seeking stage, $i=1,\ldots,n_{I}$, denoted $I_{H_{i}}(t)$. The first host-seeking stage (*i*=1) consists of exposed, host-seeking mosquitoes whose *Plasmodium* infection age reached *T_E_* at all times *t-*$\xi_{1}$ from the earliest possible time *t*-*T_E_* to the present. These mosquitoes then survived in the first host-seeking stage of the infectious stage for $\xi_{1}$ days without contracting the fungal pathogen or finding a blood meal, with probability $\theta_{I_{H}}(\xi_{1})$ (Table S1). Mosquitoes can also enter the first host-seeking stage of the infectious stage if their *Plasmodium* infection age reaches *T_E_* while they are non-host-seeking, at $a_{T_{E}}$days into the non-host-seeking stage, $0\leq a_{T_{E}}\leq T_{M}$ (Table S3). These mosquitoes then survive the remaining $T_{M}-a_{T_{E}}$days in the non-host-seeking stage without contracting the fungus, with probability $\theta_{I_{M}}(T_{M}-a_{T_{E}})$ (Table S1), and then survive in the first host-seeking stage for $\xi_{1}$ days without contracting the fungal pathogen or finding a blood meal. Thus,

$I_{H_{1}}(t)=\int_{0}^{T_{E}} \theta_{I_{H}}(\xi_{1})(E_{H}(t-\xi_{1},T_{E})+\int_{0}^{T_{M}} E_{M}(t-\xi_{1}-(T_{M}-a_{T_{E}}),T_{E},a_{T_{E}})\theta_{I_{M}}(T_{M}-a_{T_{E}})da_{T_{E}})d\xi_{i}$ (S1.12)

For host-seeking stages *i>*1 the mosquito density is those mosquitoes that found a blood meal in host-seeking stage *i*-1 at time *t-*$\xi_{i}$-*T_M_* multiplied by the probability of surviving the *i*^th^ non-host-seeking stage without contracting the fungus and then surviving the remaining $\xi_{i}$ days in $I_{H_{i}}(t)$ without finding a blood meal or contracting the fungus.

$I_{H_{i}}(t)=\int_{0}^{t-T_{E}-(i-1)T_{M}} I_{H_{i-1}}(t-\xi_{i}-T_{M})f\theta_{I_{M}}(T_{M})\theta_{I_{H}}(\xi_{i})d\xi_{i}, i>1$ (S1.13)

Now consider infectious mosquitoes in non-host-seeking stages *i*. For mosquitoes uninfected with the fungal pathogen, we track the time since their most recent blood meal, *a*, $0\leq a\leq T_{M}$ (Table S3), and denote the *i*^th^ non-host-seeking stage $I_{M_{i}}(t,a)$. The density of mosquitoes in the first non-host-seeking stage (*i*=1) is those mosquitoes whose *Plasmodium* infection age reached *T_E_* when they were $a_{T_{E}}$days through a non-host-seeking stage (Table S3). These mosquitoes then survived $a-a_{T_{E}}$days in the first non-host-seeking stage of the infectious stage without contracting the fungal pathogen.

$I_{M_{1}}(t)=\int_{0}^{T_{M}} \int_{0}^{a} E_{M}(t-(a-a_{T_{E}}),T_{E},a_{T_{E}})\theta_{I_{M}}(a-a_{T_{E}})da_{T_{E}}da$ (S1.14)

The density of mosquitoes without fungal infection in non-host-seeking stages *i*>1 is those mosquitoes that found a blood meal in host-seeking-stage *i*-1 at time *t-a* then survived *a* days in the *i*^th^ non-host-seeking stage without contracting the fungus,

$I_{M_{i}}(t)=\int_{0}^{T_{M}} I_{H_{i-1}}(t-a)f\theta_{I_{M}}(a)da$ (S1.15)

The total densities of infectious mosquitoes without fungal infection are denoted $I_{H}(t)$and $I_{M}(t)$ for host-seeking and non-host-seeking stages respectively.

$I_{H}(t)=\sum_{i}^{n_{I}} I_{H_{i}}(t)$ (S1.16)

$I_{M}(t)=\sum_{i}^{n_{I}} I_{M_{i}}(t)$ (S1.17)

*(ii) Equilibrium solution*

From equation (S1.1) it follows that

$S_{H_{1}}^{*}=\frac{\varepsilon}{f+\mu+F}$ (S1.18)

From equation (S1.2) it follows that

$S_{H_{i}}^{*}=\frac{f^{i-1}(1-bx)^{i-1}e^{-(i-1)(\mu+F)T_{M}}\varepsilon}{(\mu+f+F)^{i}}, i>1$ (S1.19)

We then have

$S_{H}^{*}=\sum_{i=1}^{n_{S}} S_{H_{i}}^{*}$ (S1.20)

*Exposed mosquitoes*

From equations (S1.6) and (S1.7) it follows that

$E_{H_{i}}^{*}(p)=S_{H}^{*}f^{i}bxe^{-i(\mu+F)T_{M}}e^{-(\mu+f+F)(p-iT_{M})}\frac{(p-iT_{M})^{i-1}}{(i-1)!}$ (S1.21)

Then, using integration by parts,

$$E_{H_{i}}^{*}=\int_{iT_{M}}^{T_{E}} E_{H_{i}}^{*}(p)dp=S_{H}^{*}f^{i}bxe^{-i(\mu+F)T_{M}}\left(\frac{1}{(\mu+f+F)^{i}}-\sum_{k=1}^{i} \frac{(T_{E}-iT_{M})^{i-k}e^{-(\mu+f+F)(T_{E}-iT_{M})}}{(i-k)!(\mu+f+F)^{k}}\right)$$

(S1.22)

We then have

$E_{H}^{*}=\sum_{i=1}^{n_{E}} E_{H_{i}}^{*}$ (S1.23)

*Infectious mosquitoes*

From equation (S1.12)

$I_{H_{1}}^{*}=\lim_{t\to\infty} \int_{0}^{t-T_{E}} E_{H}^{*}(T_{E})e^{-(f+\mu+F)\xi_{1}}d\xi_{1}+\lim_{t\to\infty} \int_{0}^{T_{M}} E_{H}^{*}(T_{E}-a)e^{-(\mu+F)(T_{M}-a)}da\int_{0}^{t-T_{E}} e^{-(f+\mu+F)\xi_{1}}d\xi_{1}$ (S1.24)

Substituting in (S1.21) and integrating by parts gives

$\begin{matrix} I_{H_{1}}^{*}=\sum_{k=1}^{n_{E}} S_{H_{k}}^{*}f^{k+1}bxe^{-(k+1)(\mu+F)T_{M}}e^{-(\mu+f+F)(T_{E}-kT_{M})}\left(\sum_{j=1}^{k} \frac{(T_{E}-(k+1)T_{M})^{k-j}e^{(\mu+f+F)T_{M}}-(T_{E}-kT_{M})^{k-j})}{(k-j)!(\mu+f+F)^{j+1}}\right. \\ +\left.\frac{(T_{E}-kT_{M})^{k-1}}{(k-1)!(\mu+f+F)}\right) \end{matrix}$ (S1.25)

From equation (S1.13) we then have

$\begin{matrix} I_{H_{i}}^{*}=\frac{I_{H_{i-1}}^{*}e^{-(\mu+F)T_{M}}f}{(\mu+f+F)}=I_{H_{1}}^{*}m^{i-1}, i>1 \\ m=\frac{e^{-(\mu+F)T_{M}}f}{(\mu+f+F)} \end{matrix}$ (S1.26)

and then

$I_{H}^{*}=\sum_{i=1}^{n_{I}} I_{H_{i}}^{*}$ (S1.27)
