# Supplementary material for: Combining Fungal Biopesticides and Insecticide-Treated Bednets to Enhance Malaria Control
Source: PLoS Comput Biol. 2009 Oct 2;5(10):e1000525. doi: 10.1371/journal.pcbi.1000525 (PMC2742557; doi:10.1371/journal.pcbi.1000525)
Supplement: Text S2 — Dynamic system and equilibria for stages of mosquitoes infected with the fungal pathogen. (0.28 MB DOC) [file pcbi.1000525.s002.docm]

Text S2: Dynamic system and equilibria for stages of mosquitoes infected with the fungal pathogen

*(i) Mosquito population dynamics*

*Susceptible mosquitoes*

We now consider susceptible mosquitoes that have been infected with the fungal pathogen for *u* days, in host-seeking stages *i,* $i=1,...,n_{S}$. Mosquitoes remain in the first host-seeking stage (*i*=1) for *u* days after contracting the fungus if they do not die due to fungal pathogen-induced mortality or other sources of mortality, and they do not find a blood meal in that time. The probability of this occurring, $\theta_{S_{HF}}(u,u)$, is defined in Table S2. The density of mosquitoes in the first host-seeking stage, denoted $S_{HF_{1}}(t,u)$, is thus given by

$S_{HF_{1}}(t,u)=S_{H_{1}}(t-u)F\theta_{S_{HF}}(u,u)$ (S2.1)

Host-seeking stages $i>1$ are denoted $S_{HF_{i}}(t,u,\xi_{i})$, where mosquitoes spend$\xi_{i}$ days in stage *i*. These stages are comprised of mosquitoes that entered via three possible routes. Firstly, fungal pathogen-infected mosquitoes that left the *i*-1^th^ host-seeking stage at time $t-T_{M_{F}}-\xi_{i}$ when their fungal infection age was $u-T_{M_{F}}-\xi_{i}$, having found a host and not contracted the *Plasmodium* (Table S3). These then survived the *i*-1^th^ non-host-seeking period, lasting $T_{M_{F}}$days, and remained in the *i*^th^ host-seeking period for time$\xi_{i}$. They have fungal infection age $u>T_{M_{F}}+\xi_{i}$. Secondly, mosquitoes that contracted the fungus during the *i-*1*^th^* non-host-seeking stage at time *t-u,* survived the rest of this non-host-seeking stage and then survived in the *i*^th^ host-seeking stage for time $\xi_{i}$ without finding a blood meal. These mosquitoes have a fungal infection age $\xi_{i}$*< u < T_M_* + $\xi_{i}$. Thirdly, mosquitoes in that contracted the fungus whilst in the *i*^th^ host-seeking stage at time *t-u* and then survived for *u* days without finding a blood meal. For these mosquitoes, $u<\xi_{i}$ . Thus

$S_{HF_{i}}(t,u,\xi_{i})=\left\{\begin{matrix} S_{H_{i}}(t-u)F\theta_{S_{HF}}(u,u), u\leq\xi_{i} \\ S_{H_{i-1}}(t-u)f\theta_{S_{M}}(T_{M}-(u-\xi_{i}))F\theta_{S_{MF}}(u-\xi_{i},u-\xi_{i})\theta_{S_{HF}}(\xi_{i},u) \xi_{i}<u\leq T_{M}+\xi_{i} \\ S_{HF_{i-1}}(t-\xi_{i}-T_{M_{F}},u-T_{M_{F}}-\xi_{i})f_{F}(1-bx)\theta_{S_{MF}}(T_{M_{F}},u-\xi_{i})\theta_{S_{HF}}(\xi_{i},u), u>T_{M_{F}}+\xi_{i}, \\ i>1 \end{matrix}\right.$ (S2.2)

where $\theta_{S_{MF}}(\cdot,\cdot)$ and $\theta_{S_{HF}}(\cdot,\cdot)$ are stage-specific probability functions defined in Table S2.

In calculating the density of non-host-seeking mosquitoes that are infected with the fungus we track both the age of the fungal infection, *u,* and the time since the most recent blood meal, *a*, $0\leq a\leq T_{M_{F}}$(Table S3). The mosquito density in non-host-seeking stage *i,* denoted $S_{MF_{i}}(t,u,a)$, consists firstly of mosquitoes that were in the *i*^th^ host-seeking stage at time *t-a* with fungal infection age *u-a*, at which point they found a blood meal and did not contract the *Plasmodium.* They then survived in the *i*^th^ non-host seeking stage for *a* days, avoiding fungal-pathogen-induced mortality and other mortality sources, with probability $\theta_{S_{MF}}(a,u)$(Table S2). Stages $S_{MF_{i}}(t,u,a)$ also consist of mosquitoes that contracted the fungus whilst in the *i*^th^ non-host-seeking stage at time *t-u*. They then survived with the fungal infection for *u* days. Thus,

$S_{MF_{1}}(t,u,a)=\left\{\begin{matrix} S_{HF_{1}}(t-a,u-a) f_{F}(1-bx)\theta_{S_{MF}}(a,u), u>a, 0\leq a\leq T_{M_{F}} \\ S_{M_{1}}(t-u,a-u)F\theta_{S_{MF}}(u,u), u\leq a, 0\leq a\leq T_{M} \end{matrix}\right.$ (S2.3)

and

$$S_{MF_{i}}(t,u,a)=\left\{\begin{matrix} \int_{0}^{\infty} S_{HF_{i}}(t-a,u-a, \xi_{i}) d\xi_{i} f_{F}(1-bx)\theta_{S_{MF}}(a,u), u>a, 0\leq a\leq T_{M_{F}} \\ S_{M_{i}}(t-u,a-u)F\theta_{S_{MF}}(u,u), u\leq a, 0\leq a\leq T_{M}, \\ i>1 \end{matrix}\right.$$

(S2.4)

The total densities of susceptible, fungal pathogen-infected mosquitoes in each stage of the gonotrophic cycle are denoted $S_{HF}(t)$and $S_{MF}(t)$ for host-seeking and non-host-seeking stages respectively.

$S_{HF}(t)=\int_{0}^{\infty} S_{HF}(t,u) du+\sum_{i=2}^{n_{S}} \int_{0}^{\infty} \int_{0}^{\infty} S_{HF_{i}}(t,u,\xi_{i}) du d\xi_{i}$ (S2.5)

$S_{MF}(t)=\sum_{i}^{n_{S}} \int_{0}^{ T_{M{}_{F}}} \int_{0}^{\infty} S_{MF_{i}}(t,u,a) du_{i} da$ (S2.6)

*Exposed mosquitoes*

For exposed, host-seeking mosquitoes that are infected with the fungal pathogen, we track both the *Plasmodium* infection age *p* and the fungal infection age *u.* The density of mosquitoes in the first host-seeking stage (*i*=1), denoted $E_{HF_{1}}(t,p,u)$, consists of mosquitoes that entered the stage from three possible sources. Firstly, from susceptible, fungal-pathogen infected mosquitoes that found a blood meal and contracted the *Plasmodium* at time *t-p* when their fungal infection age was *u-p* days. These mosquitoes survived the first non-host-seeking stage of the exposed stage, lasting $T_{M_{F}}$ days, and then avoided mortality due to fungal infection or other mortality sources for *p-*$T_{M_{F}}$ days in the first host-seeking stage, without finding a blood meal. For these mosquitoes *u*>*p.* Secondly, from susceptible mosquitoes uninfected with the fungal pathogen who found a blood meal at time *t-p* and then contracted the fungal pathogen during the first non-host-seeking stage of the exposed stage, at time *t-u.*  They then survived the remainder of this non-host-seeking stage, and survived in the first host-seeking stage for *p-*$T_{M}$ days without finding a bloodmeal. For these mosquitoes $p-T_{M}<u\leq p.$ Lastly, from exposed mosquitoes who contracted the fungal pathogen in the first host-seeking stage at time *p-u*  then survived without finding a blood meal for *u* days. For these mosquitoes $u\leq p-T_{M}.$ For all mosquitoes in $E_{HF_{1}}(t,p,u)$, $T_{M}\leq p\leq T_{E}$. Therefore,

$$E_{HF_{1}}(t,p,u)=\left\{\begin{matrix} E_{H_{1}}(t-u,p-u)F\theta_{E_{HF}}(u,u), u\leq p-T_{M} \\ S_{H}(t-p)fbx\theta_{E_{M}}(p-u)F\theta_{E_{MF}}(T_{M}-(p-u),T_{M}-(p-u))\theta_{E_{HF}}(p-T_{M},u), p-T_{M}<u\leq p \\ S_{HF}(t-p,u-p)f_{F}bx\theta_{E_{MF}}(T_{M_{F}},u-p+T_{M_{F}})\theta_{E_{HF}}(p-T_{M_{F}},u), u>p \end{matrix}\right.$$

(S2.7)

where the stage-specific survival probabilities $\theta_{E_{HF}}(\cdot,\cdot)$ and $\theta_{E_{MF}}(\cdot,\cdot)$ are defined in Table S2.

For host-seeking stages $i>1$, the density of exposed fungal-pathogen infected mosquitoes is denoted $E_{HF_{i}}(t,p,u,\xi_{i})$ where $\xi_{i}$ is the time spent in the *i*^th^ host-seeking stage (Table S3). Similar to (S2.7), mosquitoes in this stage can also come via three different routes. Firstly, from exposed mosquitoes that found a blood meal in host-seeking stage *i*-1 at time $t-T_{M_{F}}-\xi_{i}$ when their *Plasmodium* infection age was $p-T_{M_{F}}-\xi_{i}$ and their fungal infection age was $u-T_{M_{F}}-\xi_{i}$. They then survived the following non-host-seeking period, lasting $T_{M_{F}}$days, and then remained in the *i*^th^ feeding cycle for $\xi_{i}$days without finding a blood meal. For these mosquitoes, $u>T_{M_{F}}+\xi_{i}$. Secondly, from exposed mosquitoes uninfected with the fungal pathogen that found a blood meal in host-seeking stage *i*-1 at time $t-T_{M}-\xi_{i}$ when their *Plasmodium* infection age was $p-T_{M}-\xi_{i}$days. These mosquitoes then contracted the fungal infection during the *i*^th^ non-host-seeking period at time *t-u*, survived the rest of the non-host-seeking stage, and then survived for $\xi_{i}$days in the *i*^th^ host-seeking stage without finding a blood meal. For these mosquitoes, $\xi_{i}<u\leq T_{M}+\xi_{i}$. Lastly, from exposed mosquitoes that became infected with the fungus in the *i*^th^ host-seeking stage, and then survived for a further *u* days without finding a blood meal. For these mosquitoes $u\leq\xi_{i}$. For all mosquitoes in $E_{HF_{i}}(t,p,u,\xi_{i})$, $i T_{M}+\xi_{i}\leq p\leq T_{E}$. Therefore,

$E_{HF_{i}}(t,p,u,\xi_{i})=\left\{\begin{matrix} E_{H_{i}}(t-u,p-u)F\theta_{E_{HF}}(u,u), u\leq\xi_{i} \\ E_{H_{i-1}}(t-T_{M}-\xi_{i},p-T_{M}-\xi_{i})f\theta_{E_{M}}(T_{M}-(u-\xi_{i}))F\theta_{E_{MF}}(u-\xi_{i},u-\xi_{i})\theta_{E_{HF}}(\xi_{i},u), \\ \xi_{i}<u\leq T_{M}+\xi_{i} \\ E_{HF_{i-1}}(t-T_{M_{F}}-\xi_{i},p-T_{M_{F}}-\xi_{i},u-T_{M_{F}}-\xi_{i})f_{F}\theta_{E_{MF}}(T_{M_{F}},u-\xi_{i})\theta_{E_{HF}}(\xi_{i},u), \\ u>T_{M_{F}}+\xi_{i} \end{matrix}\right.$ (S2.8)

For the first non-host-seeking stage (*i*=1), the density of exposed, fungal-pathogen infected mosquitoes, denoted $E_{M_{1}}(t,p,u,a)$, consists of mosquitoes from two possible sources. Firstly from susceptible, host-seeking mosquitoes that found a blood meal and contracted the *Plasmodium* at time *t-a* when their fungal infection age was *u-a.* They then survived *a* days in the first non-host-seeking stage. For these mosquitoes,$p<T_{M_{F}}$and *u>p.* Secondly, from exposed mosquitoes that caught the fungus in the first non-host-seeking stage of the exposed stage at time *t-u.* They then survived in the first non-host-seeking stage for a further *u* days. For these mosquitoes $u\leq p$ and $p<T_{M}$*.*

$$E_{MF_{1}}\left(t,p,u,a\right)=\left\{\begin{matrix} S_{HF}(t-a,u-a)f_{F}bx\theta_{E_{MF}}(a,u), u>p, p<T_{M_{F}} \\ E_{M_{1}}(t-u,p-u,a-u)F\theta_{E_{MF}}(u,u) u\leq p, p<T_{M} \end{matrix}\right.$$

(S2.9)

Densities of fungal pathogen-infected mosquitoes in non-host-seeking stages *i*>1, denoted$E_{MF_{i}}(t,p,u,a)$, are calculated in a similar manner. $E_{MF_{i}}(t,p,u,a)$is composed firstly of mosquitoes that found a blood meal when they were exposed and in host-seeking stage *i*-1 at time *t-a* with *Plasmodium* infection age *p-a* and fungal infection age *u-a.* They then survived *a* days in non-host-seeking stage *i.* For these mosquitoes *u>a* . Secondly, mosquitoes enter $E_{MF_{i}}(t,p,u,a)$by catching the fungus in the *i*^th^ non-host-seeking stage and then surviving in this non-host-seeking stage for a further *u* days. For these mosquitoes $u\leq a$*.* For all mosquitoes in $E_{MF_{i}}(t,p,u,a)$ $(i-1)T_{M}+a<p\leq T_{E}$.

$E_{MF_{i}}\left(t,p,u,a\right)=\left\{\begin{matrix} E_{HF_{i-1}}(t-a,p-a,u-a)f_{F}\theta_{E_{MF}}(a,u), u>a, \\ E_{M_{i}}(t-u,a-u)F\theta_{E_{MF}}(u,u), u\leq a, \\ i>1 \end{matrix}\right.$ (S2.10)

Total densities of exposed mosquitoes infected with the fungal pathogen are denoted $E_{HF}(t)$and $E_{MF}(t)$for host-seeking and non-host-seeking stages respectively.

$E_{HF}(t)=\int_{0}^{T_{E}} \int_{0}^{\infty} E_{HF_{1}}(t,p,u) du dp+\sum_{i=2}^{n_{S}} \int_{0}^{T_{E}} \int_{0}^{p-iT_{M}} \int_{0}^{\infty} E_{HF_{i}}(t,p,u,\xi_{i}) du d\xi_{i} dp$ (S2.11)

$E_{HF}(t)=\sum_{i}^{n_{S}} \int_{0}^{T_{M}} \int_{0}^{T_{E}} \int_{0}^{\infty} E_{HF_{i}}(t,p,u,a) du dp da$ (S2.12)

*Infectious mosquitoes*

The density of infectious mosquitoes in each host-seeking stage *i* that are infected with the fungal pathogen, denoted $I_{HF_{i}}(t,u,\xi_{i})$, is calculated for each fungal infection age *u* and time spent in the *i*^th^ host seeking stage, $\xi_{i}$. Starting with the first host-seeking stage (*i*=1), mosquitoes in $I_{HF_{1}}(t,u,\xi_{i})$ can come from four possible sources. Firstly, from fungus-infected host-seeking mosquitoes whose *Plasmodium* infection age reached *T_E_* when their fungal infection age was *u-* $\xi_{1}$ at all times *t-*$\xi_{1}$. These mosquitoes then avoided mortality due to fungal infection and other mortality sources for $\xi_{1}$ days without finding a blood meal, with probability $\theta_{I_{HF}}(\xi_{1},u)$ (Table S2). Secondly, from infectious mosquitoes that contracted the fungal pathogen during the first host-seeking stage of the infectious stage, then survived for a further *u* days without finding a blood meal. Thirdly, from non-host-seeking, fungal-pathogen infected mosquitoes whose *Plasmodium* infection age reaches *T_E_* at $a_{T_{E}}$days through a non-host-seeking stage. They then survived the remaining $T_{M}-a_{T_{E}}$days in this non-host-seeking stage, with probability $\theta_{I_{MF}}(u-\xi_{1},T_{M_{F}}-a_{T_{E}})$ (Table S2), and go on to survive $\xi_{1}$ days in the first host-seeking stage without finding a blood meal. Lastly, from non-host-seeking mosquitoes uninfected with the fungal pathogen whose *Plasmodium* infection age reaches *T_E_* at $a_{T_{E}}$days through a non-host-seeking stage. These mosquitoes then survive the remaining $T_{M}-a_{T_{E}}$days in this non-host-seeking stage, contracting the fungal pathogen at some point $a=T_{M}-(u-\xi_{1})$ through this non-host-seeking stage, where $a>a_{T_{E}}$. They then survive $\xi_{1}$ days in the first host-seeking stage without finding a blood meal. Thus,

$I_{HF_{1}}(t,u,\xi_{1})=\left\{\begin{matrix} I_{H_{1}}(t-u)F\theta_{I_{HF}}(u,u), u\leq\xi_{1} \\ E_{HF}(t-\xi_{1},T_{E},u-\xi_{1})\theta_{I_{HF}}(\xi_{1},u)+ \\ \int_{0}^{T_{M}} E_{MF}(t-\xi_{1}-(T_{M_{F}}-a_{T_{E}}),T_{E},u-\xi_{1}-(T_{M_{F}}-a_{T_{E}}),a_{T_{E}})\theta_{I_{MF}}(T_{M_{F}}-a_{T_{E}},u-\xi_{1}) da_{T_{E}} \theta_{I_{HF}}(\xi_{1},u), \\ u>\xi_{1}+(T_{M_{F}}-a_{T_{E}}) \\ \int_{0}^{T_{M}} E_{M}(t-\xi_{1}-(T_{M}-a_{T_{E}}),T_{E},a_{T_{E}})\theta_{I_{M}}(T_{M}-(u-\xi_{1})-a_{T_{E}})F\theta_{I_{MF}}(u-\xi_{1},u-\xi_{1})\theta_{I_{HF}}(\xi_{1},u), \\ \xi_{1}<u\leq\xi_{1}+(T_{M}-a_{T_{E}}) \end{matrix}\right.$ (S2.13)

Mosquitoes in host-seeking stages *i*>1 come from three possible sources. Firstly, fungal pathogen-infected mosquitoes that found a blood meal in host-seeking stage *i*-1 at time $t-T_{M_{F}}-\xi_{i}$when their fungal infection age was $u-T_{M_{F}}-\xi_{i}$, survived the *i*^th^ non-host-seeking period then survived for $\xi_{i}$ days in the *i*^th^ non-host-seeking stage without finding a blood meal. Secondly, mosquitoes uninfected with the fungal pathogen that found a blood meal at time $t-T_{M}-\xi_{i}$and contracted the fungus during the *i*^th^ non-host-seeking stage at time *t-u.* They then survived the rest of the *i*^th^ non-host-seeking stage, and survived for $\xi_{i}$ days in the *i*^th^ non-host-seeking stage without finding a blood meal. Lastly, from mosquitoes that contracted the fungus in the *i*^th^ host-seeking stage at time *t-u*, then survived for *u* days without finding a blood meal.

$$I_{HF_{i}}(t,u,\xi_{i})=\left\{\begin{matrix} I_{H_{i}}(t-u)F\theta_{I_{HF}}(u,u), u\leq\xi\\ I_{M_{i}}(t-u)F\theta_{I_{MF}}(u-\xi_{i},u-\xi_{i})\theta_{I_{HF}}(\xi_{i},u) \xi_{i}<u\leq T_{M}+\xi_{i} \\ I_{HF_{i-1}}(t-\xi_{i}-T_{M_{F}},u-T_{M_{F}}-\xi_{i})f_{F}\theta_{I_{MF}}(T_{M_{F}},u-\xi_{i})\theta_{I_{HF}}(\xi_{i},u), u>T_{M_{F}}+\xi_{i} \end{matrix}\right.$$

(S2.14)

To calculate the density of infectious, non-host-seeking mosquitoes that are fungus infected, keep track of the time since the most recent blood meal, *a* (Table S3). The density of mosquitoes in the first non-host-seeking stage (*i*=1), denoted $I_{MF_{1}}(t,u,a)$, consists of mosquitoes from two different sources. Firstly, from fungal-pathogen infected, non-host-seeking mosquitoes whose *Plasmodium* infection age reached *T_E_* at point $a_{T_{E}}$in a non-host-seeking stage (Table S3). These mosquitoes then survived a further $a-a_{T_{E}}$days in the first non-host-seeking stage of the infectious stage, with probability $\theta_{I_{MF}}(a-a_{T_{E}},u)$ (Table S2). Secondly, from non-host-seeking mosquitoes without fungal infection whose *Plasmodium* infection age reached *T_E_* at point $a_{T_{E}}$in a non-host-seeking stage. These mosquitoes then survived a further $a-a_{T_{E}}$days in the first non-host-seeking stage of the infectious stage, contracting the fungal infection at time *t-u*, where $u\leq a-a_{T_{E}}$. Thus,

$I_{MF_{1}}(t,u,a)=\left\{\begin{matrix} \int_{0}^{a} E_{MF}(t-(a-a_{T_{E}}),T_{E},u-(a-a_{T_{E}}),a_{T_{E}})\theta_{I_{MF}}(a-a_{T_{E}},u)da_{T_{E}}, u>a-a_{T_{E}}, 0\leq a\leq T_{M_{F}} \\ \int_{0}^{a} E_{M}(t-(a-a_{T_{E}}),T_{E},a_{T_{E}})\theta_{I_{M}}(a-a_{T_{E}}-u)F\theta_{I_{MF}}(u,u)da_{T_{E}}, u<a-a_{T_{E}}, 0\leq a\leq T_{M} \end{matrix}\right.$ (S2.15)

Mosquito densities in non-host-seeking stages *i>*1, denoted $I_{MF_{i}}(t,u,a)$, consist firstly of fungal pathogen-infected mosquitoes that found a blood meal at time *t-a* in the *i-*1^th^ host-seeking stage when their fungal infection age was *u-a*, and then survived *a* days in the *i*^th^ non-host-seeking stage. Secondly, infectious mosquitoes that are without fungal infection and in the *i*^th^ non-host-seeking stage enter $I_{MF_{i}}(t,u,a)$ by contracting the fungal pathogen at time *t-u* and then surviving a further *u* days, $u\leq a$*.* Thus,

$I_{MF_{i}}(t,u,a)=\left\{\begin{matrix} I_{HF_{i-1}}(t-a,u-a)f_{F}\theta_{I_{MF}}(a,u), u>a, 0\leq a\leq T_{M_{F}} \\ I_{M_{i}}(t-u,a-u)F\theta_{I_{MF}}(u,u), u\leq a, 0\leq a\leq T_{M} \end{matrix}\right.$ (S2.16)

The total densities of malaria infectious mosquitoes infected with the fungal pathogen are denoted $I_{HF}(t)$and $I_{MF}(t)$for host-seeking and non-host-seeking stages respectively.

$I_{HF}(t)=\sum_{i}^{n_{S}} \int_{0}^{\infty} \int_{0}^{\infty} I_{HF_{i}}(t,u,\xi_{i}) du d\xi_{i}$ (S2.17)

$I_{MF}(t)=\sum_{i}^{n_{S}} \int_{0}^{T_{M{}_{F}}} \int_{0}^{\infty} I_{MF_{i}}(t,u,a) du da$ (S2.18)

*(ii) Equilibrium solution*

Expressions for the equilibrium density of susceptible, exposed and infectious host-seeking mosquitoes infected with the fungal pathogen are derived for the limiting case in which $f=f_{F}$ , $T_{M}=T_{M_{F}}$and $\beta=1$. For the case where the shape parameter $\beta=1$, fungal infection has a constant linear effect on the mortality rate *μ*, so that $M_{F}(u)=\mu_{w}u$. Thus define a constant parameter *c,*  $c=\mu_{w}+1$, so that *M_F_*(*u*) + *μ=c μ*.

*Susceptible mosquitoes*

Beginning with the equilibrium density of susceptible, fungus-infected mosquitoes in the first host-seeking stage, denoted $S_{HF_{1}}^{*}$, substitute *M_F_*(*u*) + *μ=c μ* into equation (S2.1) to give

$S_{HF_{1}}^{*}=\int_{0}^{\infty} S_{H_{1}}^{*}Fe^{-(c\mu+f)u}du=\frac{S_{H_{1}}^{*}F}{c\mu+f}$ (S2.19)

The equilibrium densities in host-seeking stages *i*>1 consists of all mosquitoes that became infected with the fungal pathogen in either host-seeking stages *m*=1,…,*i* or non-host-seeking stages *m*=1,…,*i*-1. These mosquitoes then survived to take enough blood meals to enter the *i^th^* host-seeking stage. First, denote the equilibrium density of susceptible, host-seeking mosquitoes in host-seeking stage *i* that caught the fungal infection in host-seeking stage *m* as $S_{H_{im}}^{*}$. These mosquitoes survived with the fungal infection for $u\geq(i-m)T_{M}+\xi_{m+1}+\ldots+\xi_{i}$ days, progressing through host-seeking stages *m,…,i* and non-host-seeking stages *m,…,i*-1, taking *i-m* blood meals without contracting the *Plasmodium.* Since the probability of surviving host-seeking stages *m,…,i* is

$\prod_{k=m}^{i} \int_{0}^{\infty} e^{-(c\mu+f)\xi_{k}}d\xi_{k}=\frac{1}{(c\mu+f)^{i-m+1}}$ (S2.20)

and the probability of surviving non-host-seeking stages *m,…*,*i*-1 is $e^{-(i-m)c\mu T_{M}}$, we have

$S_{H_{im}}^{*}=\frac{S_{H_{m}}^{*}f^{i-m}(1-bx)^{i-m}Fe^{-(i-m)c\mu T_{M}}}{(c\mu+f)^{i-m+1}}, 1\leq m\leq i$ (S2.21)

Now denote the equilibrium density of susceptible, host-seeking mosquitoes in host-seeking stage *i* that caught the fungal infection in non-host-seeking stage *m* as $S_{M_{im}}^{*}$. For these mosquitoes we need to consider at which point in the non-host-seeking stage the mosquito contracts the fungus (denoted *a*).

$$\begin{matrix} S_{M_{im}}^{*}=\frac{S_{H_{m}}^{*}f^{i-m}(1-bx)^{i-m}Fe^{-(i-m-1)c\mu T_{M}}}{(c\mu+f)^{i-m}}\int_{0}^{T_{M}} e^{-(\mu+F)a}e^{-c\mu(T_{M}-a)}da \\ \\ =\frac{S_{H_{m}}^{*}f^{i-m}(1-bx)^{i-m}Fe^{-(i-m)c\mu T_{M}}(1-e^{-(\mu+F-c\mu)T_{M}})}{(c\mu+f)^{i-m}(\mu+F-c\mu)}, 1\leq m\leq i-1 \end{matrix}$$

(S2.22)

We now have

$S_{HF_{i}}^{*}=\sum_{m=1}^{i} S_{H_{im}}^{*}+\sum_{m=1}^{i-1} S_{H_{im}}^{*}, i>1$ (S2.23)

Similar, but more complex, expressions can be derived for $E_{HF_{i}}^{*}$and $I_{HF_{i}}^{*}$, the equilibrium densities of fungal pathogen-infected exposed and infectious host-seeking mosquitoes respectively.
