# Supplementary material for: Combining Fungal Biopesticides and Insecticide-Treated Bednets to Enhance Malaria Control
Source: PLoS Comput Biol. 2009 Oct 2;5(10):e1000525. doi: 10.1371/journal.pcbi.1000525 (PMC2742557; doi:10.1371/journal.pcbi.1000525)
Supplement: Table S1 — The functions,.θ[·], for the probabilities that mosquitoes remain in a given stage for a certain time period, for stages of mosquitoes uninfected with the fungal pathogen. (0.06 MB DOC) [file pcbi.1000525.s003.doc]

Table S1. The functions, $\theta[\cdot]$, for the probabilities that mosquitoes remain in a given stage for a certain time period, for stages of mosquitoes uninfected with the fungal pathogen.

| Function | Definition | Description |
| --- | --- | --- |
| $\theta_{S_{H}}(x)$ | $\exp[-(\mu_{F}+F+f)x]$ | Probability of remaining in a susceptible, host-seeking stage for *x* days without contracting the fungal pathogen |
| $\theta_{S_{M}}(x)$ | $\exp[-(\mu_{M}+F)x], 0\leq x\leq T_{M}$ | Probability of remaining in a susceptible, non-host-seeking stage for *x* days without contracting the fungal pathogen |
| $\theta_{E_{H}}(x)$ | $\begin{matrix} \exp[-(\mu_{F}+F+f)x], \\ T_{M}\leq x\leq T_{E} \end{matrix}$ | Probability of remaining in an exposed, host-seeking stage for *x* days without contracting the fungal pathogen |
| $\theta_{E_{M}}(x)$ | $\exp[-(\mu_{M}+F)x], 0\leq x\leq T_{M}$ | Probability of remaining in an exposed, non-host-seeking stage for *x* days without contracting the fungal pathogen |
| $\theta_{I_{H}}(x)$ | $\exp[-(\mu_{F}+F+f)x]$ | Probability of remaining in an infectious, host-seeking stage for *x* days without contracting the fungal pathogen |
| $\theta_{I_{M}}(x)$ | $\exp[-(\mu_{M}+F)x], 0\leq x\leq T_{M}$ | Probability of remaining in an infectious, non-host-seeking stage for *x* days without contracting the fungal pathogen |
