# Supplementary material for: Combining Fungal Biopesticides and Insecticide-Treated Bednets to Enhance Malaria Control
Source: PLoS Comput Biol. 2009 Oct 2;5(10):e1000525. doi: 10.1371/journal.pcbi.1000525 (PMC2742557; doi:10.1371/journal.pcbi.1000525)
Supplement: Table S2 — The functions,.θ[·], for the probabilities that mosquitoes remain in a given stage for a certain time period, for stages of mosquitoes infected with the fungal pathogen. (0.07 MB DOC) [file pcbi.1000525.s004.doc]

Table S2. The functions, $\theta[\cdot]$, for the probabilities that mosquitoes remain in a given stage for a certain time period, for stages of mosquitoes infected with the fungal pathogen.

| Function | Definition | Description |
| --- | --- | --- |
| $\theta_{S_{HF}}(x,y)$ | $\exp[-\int_{y-x}^{y} \mu_{F}+M_{F}(u)+f_{F} du]$ | Probability that mosquitoes remain in a susceptible, host-seeking stage for *x* days given that they have carried the fungal infection for *y* days |
| $\theta_{S_{MF}}(x,y)$ | $\begin{matrix} \exp[-\int_{y-x}^{y} \mu_{M}+M_{F}(u) du], \\ 0\leq x\leq T_{M_{F}} \end{matrix}$ | Probability that mosquitoes remain in a susceptible, non-host-seeking stage for *x* days given that they have carried the fungal infection for *y* days |
| $\theta_{E_{HF}}(x,y)$ | $\begin{matrix} \exp[-\int_{y-x}^{y} \mu_{F}+M_{F}(u)+f_{F} du], \\ T_{M}\leq x\leq T_{E} \end{matrix}$ | Probability that mosquitoes remain in an exposed, host-seeking stage for *x* days given that they have carried the fungal infection for *y* days |
| $\theta_{E_{MF}}(x,y)$ | $\begin{matrix} \exp[-\int_{y-x}^{y} \mu_{M}+M_{F}(u) du], \\ 0\leq x\leq T_{M_{F}} \end{matrix}$ | Probability that mosquitoes remain in an exposed, non-host-seeking stage for *x* days given that they have carried the fungal infection for *y* days |
| $\theta_{I_{HF}}(x,y)$ | $\exp[-\int_{y-x}^{y} \mu_{F}+M_{F}(u)+f_{F} du]$ | Probability that mosquitoes remain in an infectious, host-seeking stage for *x* days given that they have carried the fungal infection for *y* days |
| $\theta_{I_{MF}}(x,y)$ | $\begin{matrix} \exp[-\int_{y-x}^{y} \mu_{M}+M_{F}(u) du], \\ 0\leq x\leq T_{M_{F}} \end{matrix}$ | Probability that mosquitoes remain in an infectious, non-host-seeking stage for *x* days given that they have carried the fungal infection for *y* days |
