# Supplementary material for: Combining Fungal Biopesticides and Insecticide-Treated Bednets to Enhance Malaria Control
Source: PLoS Comput Biol. 2009 Oct 2;5(10):e1000525. doi: 10.1371/journal.pcbi.1000525 (PMC2742557; doi:10.1371/journal.pcbi.1000525)
Supplement: Table S3 — Model variables. (0.05 MB DOC) [file pcbi.1000525.s005.doc]

Table S3. Model variables

| Symbol | Definition | Value |
| --- | --- | --- |
| *p* | age of *Plasmodium* infection | 0-*T_E_* (d) |
| *u* | age of fungal infection | varies (d) |
| *a* | time that non-host-seeking mosquitoes have spent in the current non-host-seeking stage | varies (d) |
| $a_{T_{E}}$ | time that non-host-seeking mosquitoes have spent in the current non-host-seeking stage when their *Plasmodium* infection age reaches *T_E_* | varies (d) |
| $\xi_{i}$ | time spent in the *i*^th^ host-seeking stage | varies (d) |
| $n_{S}$ | number of gonotrophic cycles beyond which the probability of remaining in the susceptible stage is less than 0.0001. | 10 |
| $n_{E}$ | maximum number of gonotrophic cycles in the exposed stage | $\left\lceil T_{E}/T_{M}\right\rceil$ |
| $n_{I}$ | number of gonotrophic cycles beyond which the probability of remaining in the infectious stage is less than 0.0001. | 10 |
| $\mu_{w}$ | Rate parameter of the Weibull model of fungal pathogen-induced mortality (see text) | varies |
| *β* | Shape parameter of the Weibull model of fungal pathogen-induced mortality (see text) | 3.5 |
| $M_{F}(u)$ | Fungal pathogen-induced mortality rate | varies |
| $g_{F}$ | Average time to death due to fungal infection (see text) | varies |
